# Supplementary figures and images for: Nematocida displodere mechanosensitive ion channel of small conductance 2 assembles into a unique 6-channel super-structure in vitro
Source: PLoS One. 2024 Jul 22;19(7):e0301951. doi: 10.1371/journal.pone.0301951 (PMC11262690; doi:10.1371/journal.pone.0301951)

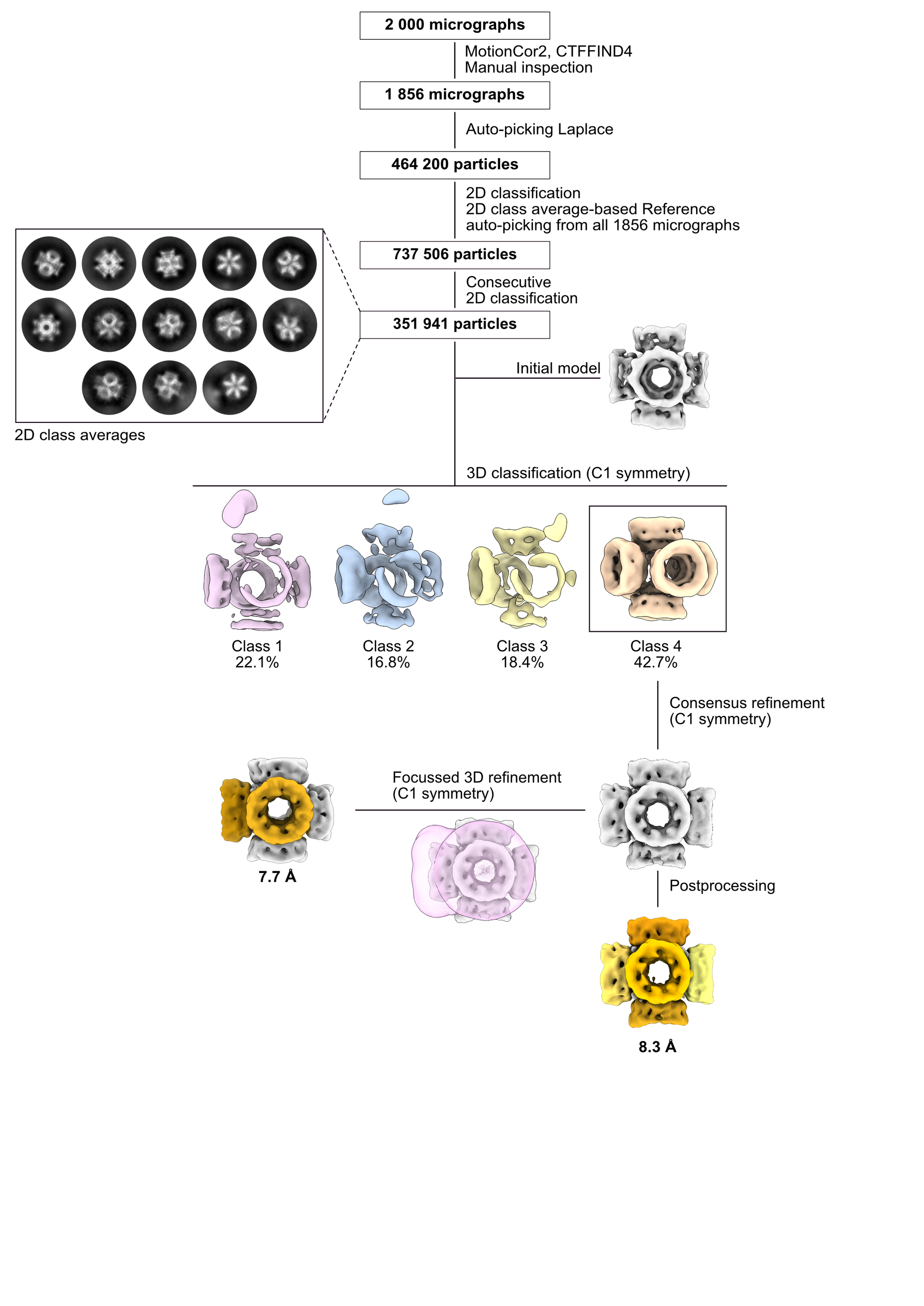

Supplement: S1 Fig — Initially, 2000 micrographs were collected and manually inspected to remove any affected by drift, poor CTF fits, or low-quality ice, resulting in a total of 1856 micrographs. Particles were picked through Laplacian auto-picking, followed by 2D classification and 2D class average-based template picking on all 1856 micrographs. Consecutive 2D classification was used to eliminate picking contaminants and sort out suboptimal classes. An initial model was generated from the resulting 351,941 particles, and a 3D classification with image alignment was performed to find the most populated state. Particles contributing to this state underwent subsequent consensus refinement without symmetry, followed by post-processing, which yielded an 8.3 Å volume. To obtain an improved single-channel volume, focused refinement was performed on the two best-looking channels, leading to a 7.7 Å volume more clearly indicating a homo-heptameric assembly per MscS2 channel. (TIF) [file pone.0301951.s001.tif]

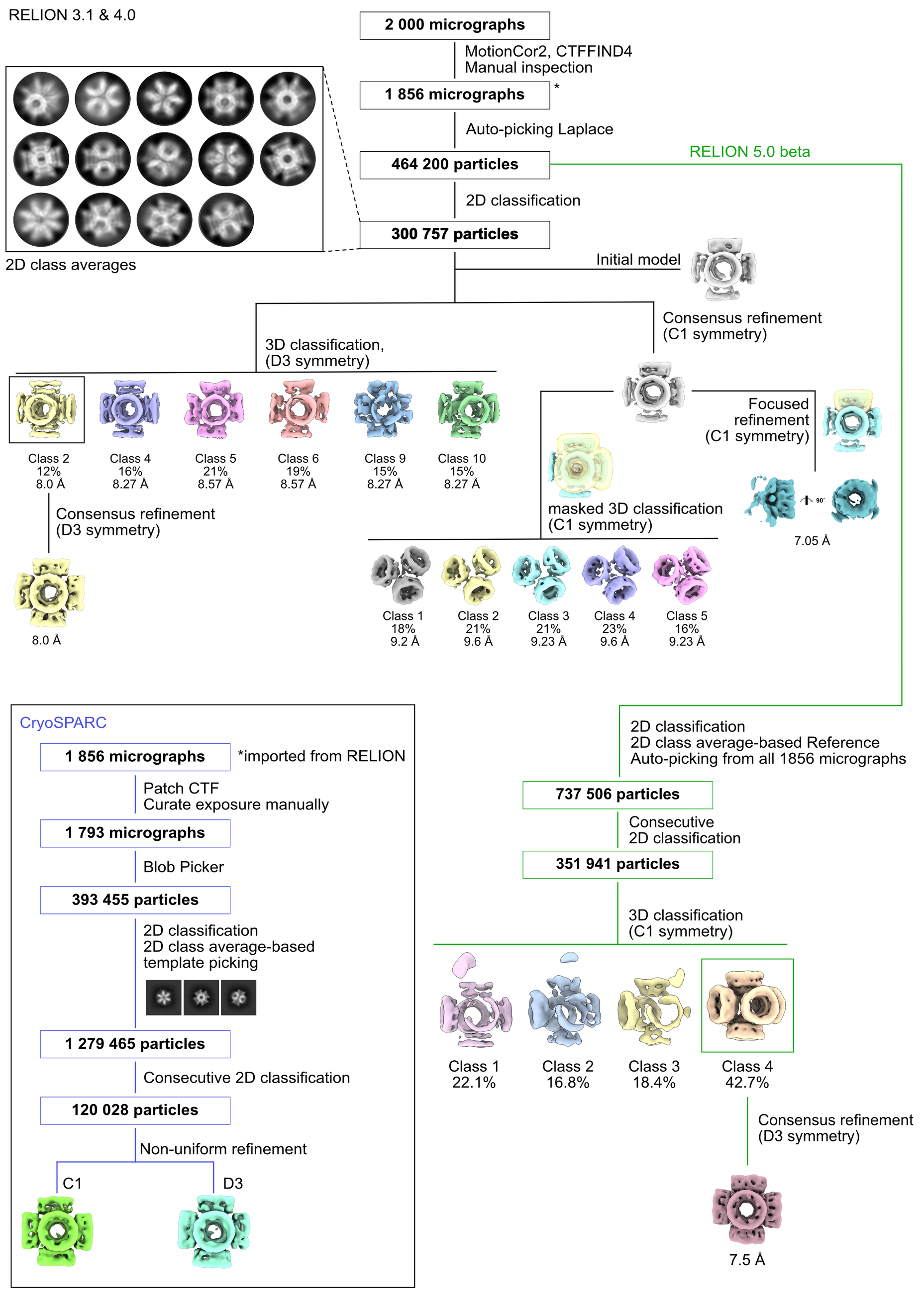

Supplement: S2 Fig — Initially, 2000 micrographs were collected and manually inspected in RELION 3.1 and 4.0 (black branch) to remove any affected by drift, poor CTF fits, or low-quality ice, resulting in a total of 1856 micrographs. Particles were picked through Laplacian auto-picking and underwent one round of 2D classification to eliminate picking contaminants. An initial model was generated from the resulting 300,757 particles. A subsequent 3D classification with D3 symmetry, followed by a consensus refinement of particles in the best class, led to an 8 Å volume lacking connectivity and proper features. Consensus refinement of the 300,757 particles without symmetry, followed by post-processing, yielded an 8.3 Å volume. Focused refinement of a single heptamer led to a featureless 7 Å volume. 3D classification of three heptamers in the C1 refined volume gave no additional information. Later, RELION 5.0 beta (green branch) was used to generate 2D class averages for reference-based picking from all 1856 micrographs. The extracted particles were subjected to multiple rounds of 2D classification to sort out suboptimal classes, followed by 3D classification. Particles of the most populated class 4 underwent consensus refinement with D3 symmetry, resulting in a 7.5 Å volume. However, the features are not as pronounced as in our published volume. Next, the 1856 motion-corrected micrographs were imported into CryoSPARC (blue branch), and CTF estimation and manual exposure correction were performed. From the resulting 1793 micrographs, 393 455 particles were picked and subjected to 2D classification for 2D-class average-based template generation. Template picking from the 1793 micrographs yielded 1,279,465 particles. Picking contaminants and suboptimal classes were filtered through consecutive 2D classification, resulting in 120,028 particles subjected to both C1 and D3 symmetry-based non-uniform refinement, respectively. The obtained volumes had incomplete features and showed insufficient con [file pone.0301951.s002.tif]

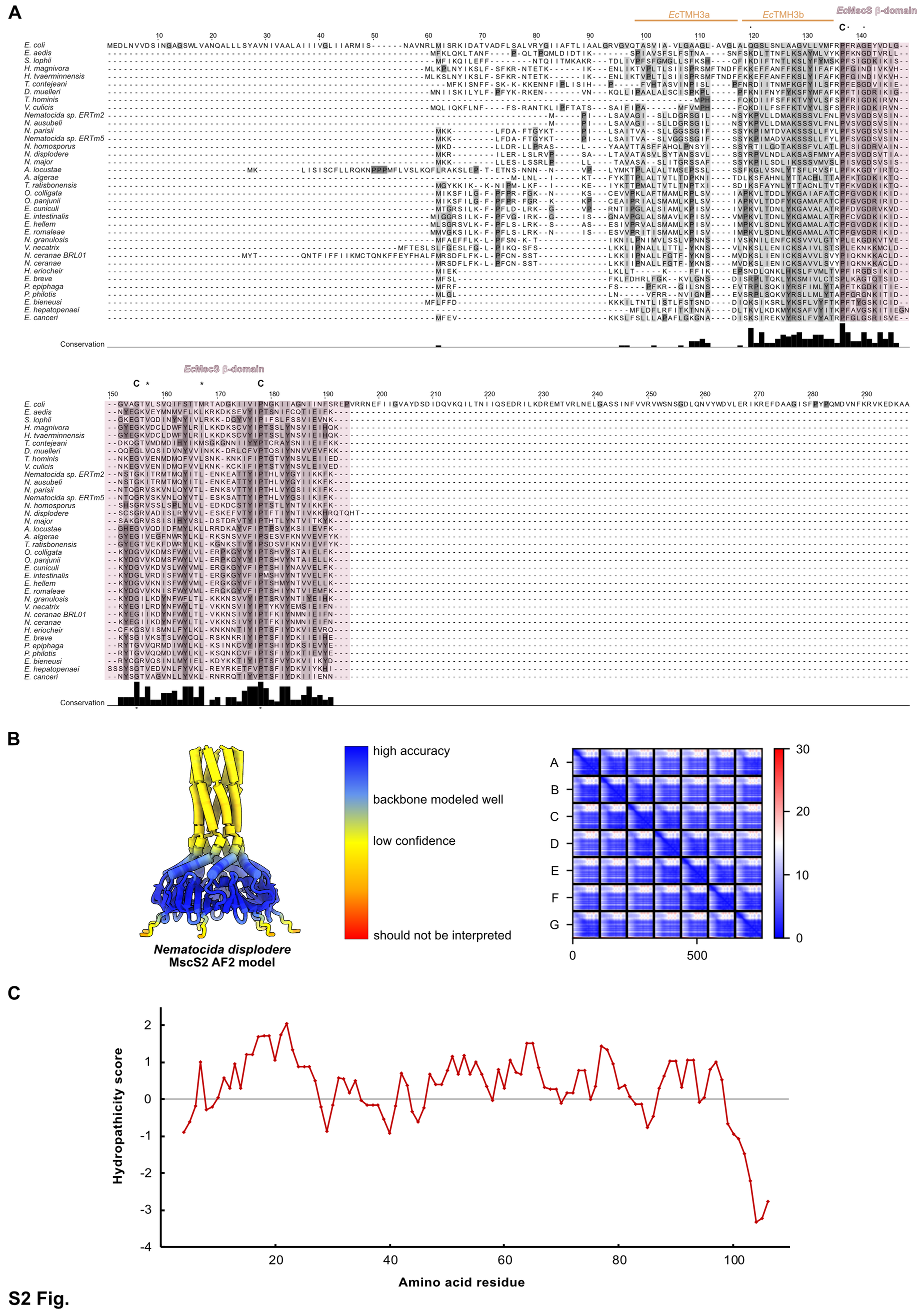

Supplement: S3 Fig — A) Multiple sequence alignment (MSA) of E. coli MscS and microsporidian MscS2 shows high conservation of the MscS β-domain and moderate conservation of the transmembrane helix 3b (TMH3b). MSA was generated using ClustalW [42] with default settings, colored according to conservation with “Clustal” in Jalview (v2.11.2.6) and set to black and white. B) AlphaFold2 (v2.3.1) prediction of homo-heptameric N. displodere MscS2 (left), colored by pLDDT confidence measure, with indicated color key, and predicted aligned error plots (right). C) Hydropathicity prediction for N. displodere MscS2 to identify hydrophobic regions, calculated using ProtScale on the ExPASy server [49]. (TIF) [file pone.0301951.s003.tif]

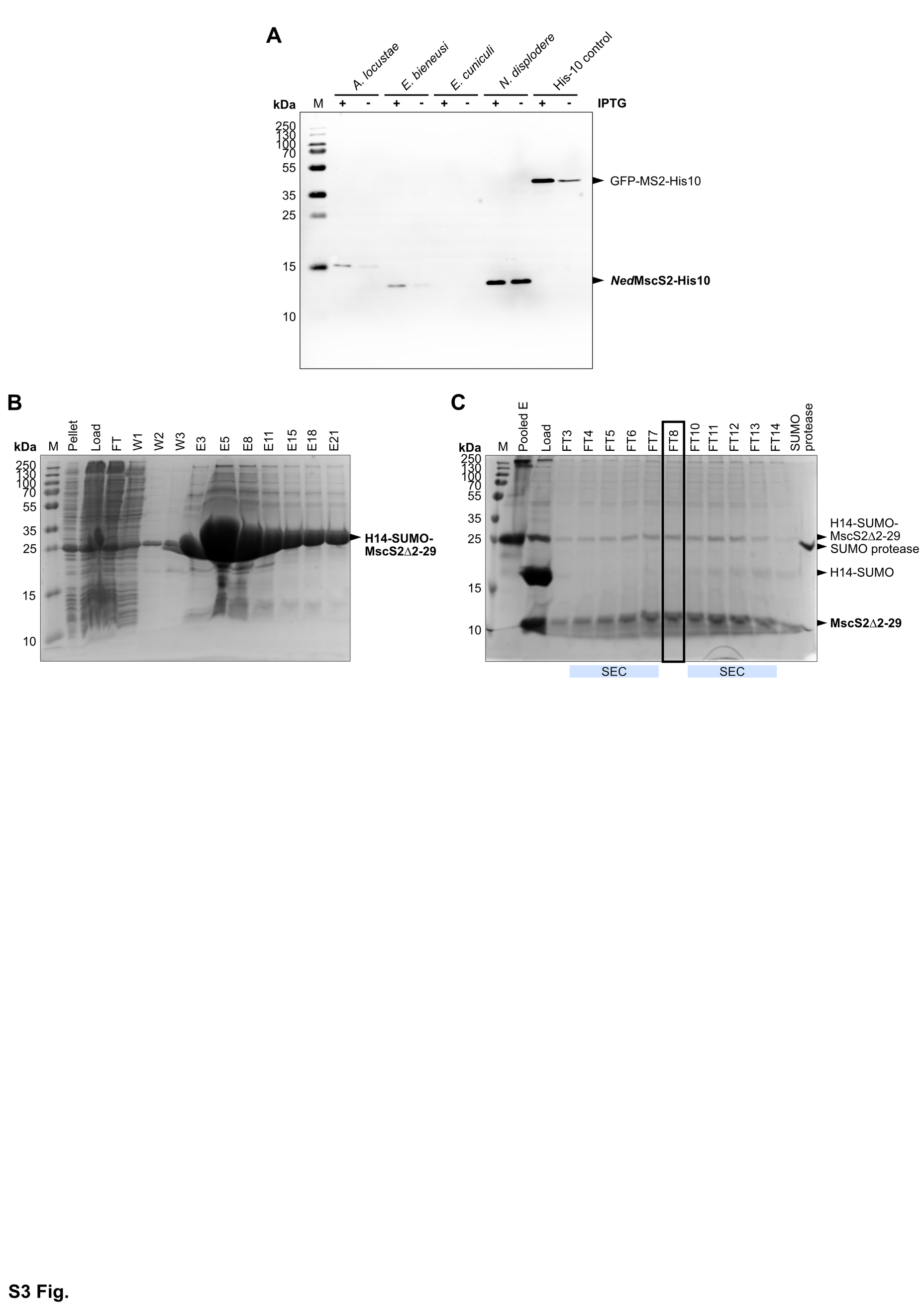

Supplement: S4 Fig — A) Western Blot analysis of induced and non-induced cultures comparing MscS2 yields from A. locustae, E. bieneusi, E. cuniculi, and N. displodere produced with a C-terminal His10 tag in E. coli Rosetta (DE3). Anti-His6x antibody from mouse was used for immunoprecipitation (1:1000). GFP-MS2-His10 construct served as positive control for protein production and antibody binding. As seen in the blots, the promotor is leaky as MscS2 is produced to some extent in the absence of IPTG. B) IMAC purification of DDM-solubilized His14-SUMO-MscS2Δ2–29 visualized by SDS-PAGE showing that the protein can be produced in high amounts. C) SDS-PAGE analysis of the fractions corresponding to the reverse IMAC of DDM-solubilized MscS2Δ2–29 post tag cleavage. Fraction used for negative-stain TEM studies is boxed in black and fractions analyzed via SEC are marked with blue boxes. kDa, kilo Dalton; M, marker; IPTG, Isopropyl β-d-1-thiogalactopyranoside; GFP, green-fluorescent protein; MS2, MS2 bacteriophage coat protein; pooled E, pooled elution; FT, flowthrough; SEC, size exclusion chromatography, kDa, kilo Dalton; M, marker; FT, flowthrough; W, wash; E, elution; SUMO, small ubiquitin-related modifier; SEC, size-exclusion chromatography. (TIF) [file pone.0301951.s004.tif]

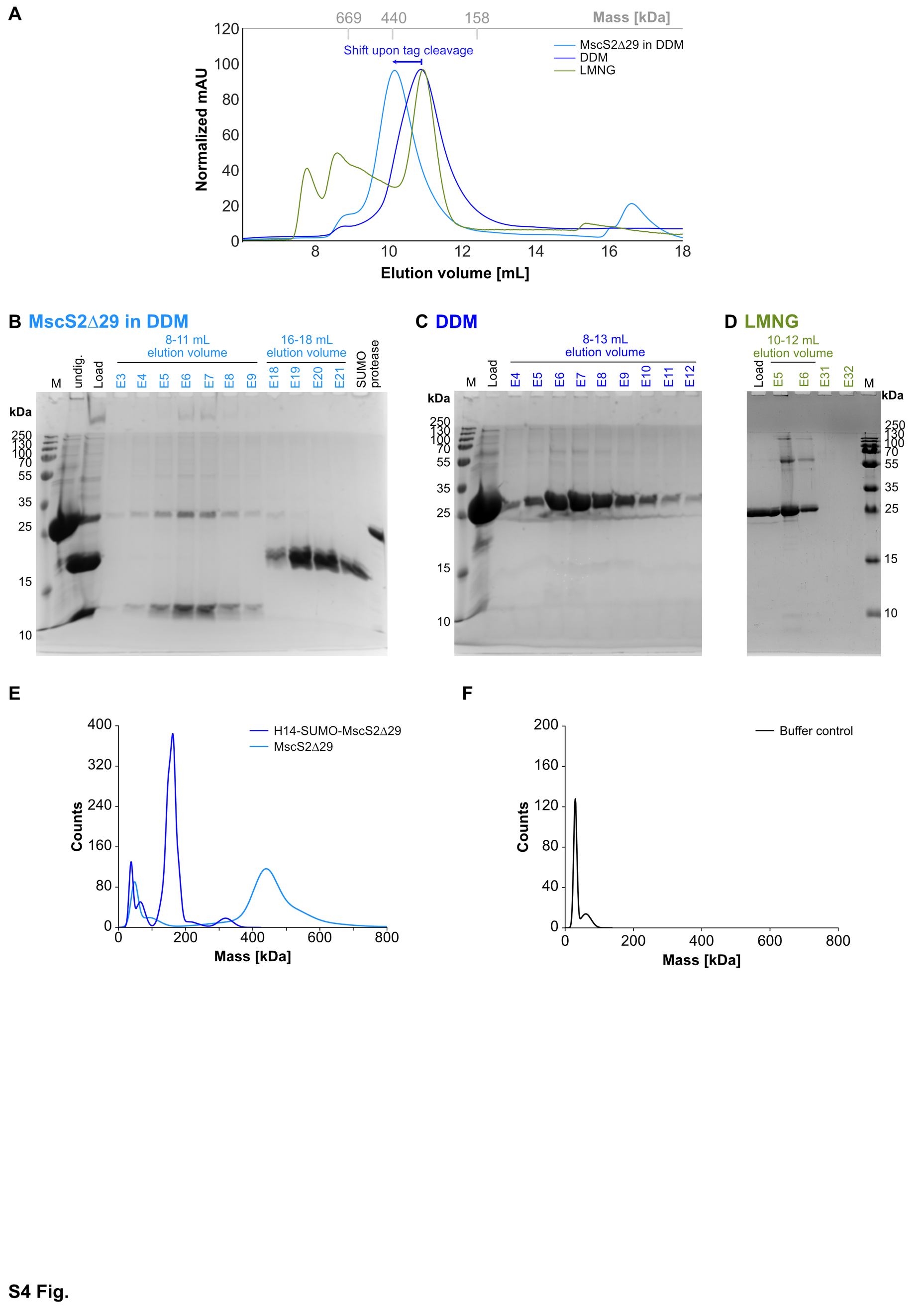

Supplement: S5 Fig — A) Size exclusion chromatogram (SEC) profile of DDM and LMNG-solubilized MscS2Δ2–29 with and without (only for DDM) His14-SUMO tag. DDM-solubilized MscS2Δ2–29 elutes at 10.16 mL, His14-SUMO-MscS2Δ2–29 in DDM elutes at 10.87, and the latter solubilized with LMNG elutes at 10.93 mL. The observed main peak shift post tag cleavage is indicated. The x-axis of the SEC profile shows the elution volume in milliliters (mL), the y-axis displays the absorption in milli-absorbance units, and a second x-axis at the top shows the molecular weight of standard globular proteins. B-D) SDS-PAGE analyses of the indicated SEC-elution fractions for B) MscS2Δ2–29 (ca. 9 kDa) and C) His14-SUMO-MscS2Δ2–29 (ca. 23 kDa), both solubilized using DDM and D) His14-SUMO-MscS2Δ2–29 solubilized with LMNG. The main SEC peaks in A) correspond to MscS2Δ2–29 with or without His14-SUMO tag. E) Mass photometry analysis comparing the molecular weight of MscS2Δ2–29 with and without His14-SUMO tag. For His14-SUMO-MscS2Δ2–29 (12nM), a main peak corresponding to 158 (± 15.6) kDa was detected, and MscS2Δ2–29 (140 nM) produced a main peak at 452 (± 112) kDa. F) Buffer control of the mass photometry experiments showing a peak around 30 (± 4.8) kDa and 61 (± 15.6) kDa. (TIF) [file pone.0301951.s005.tif]

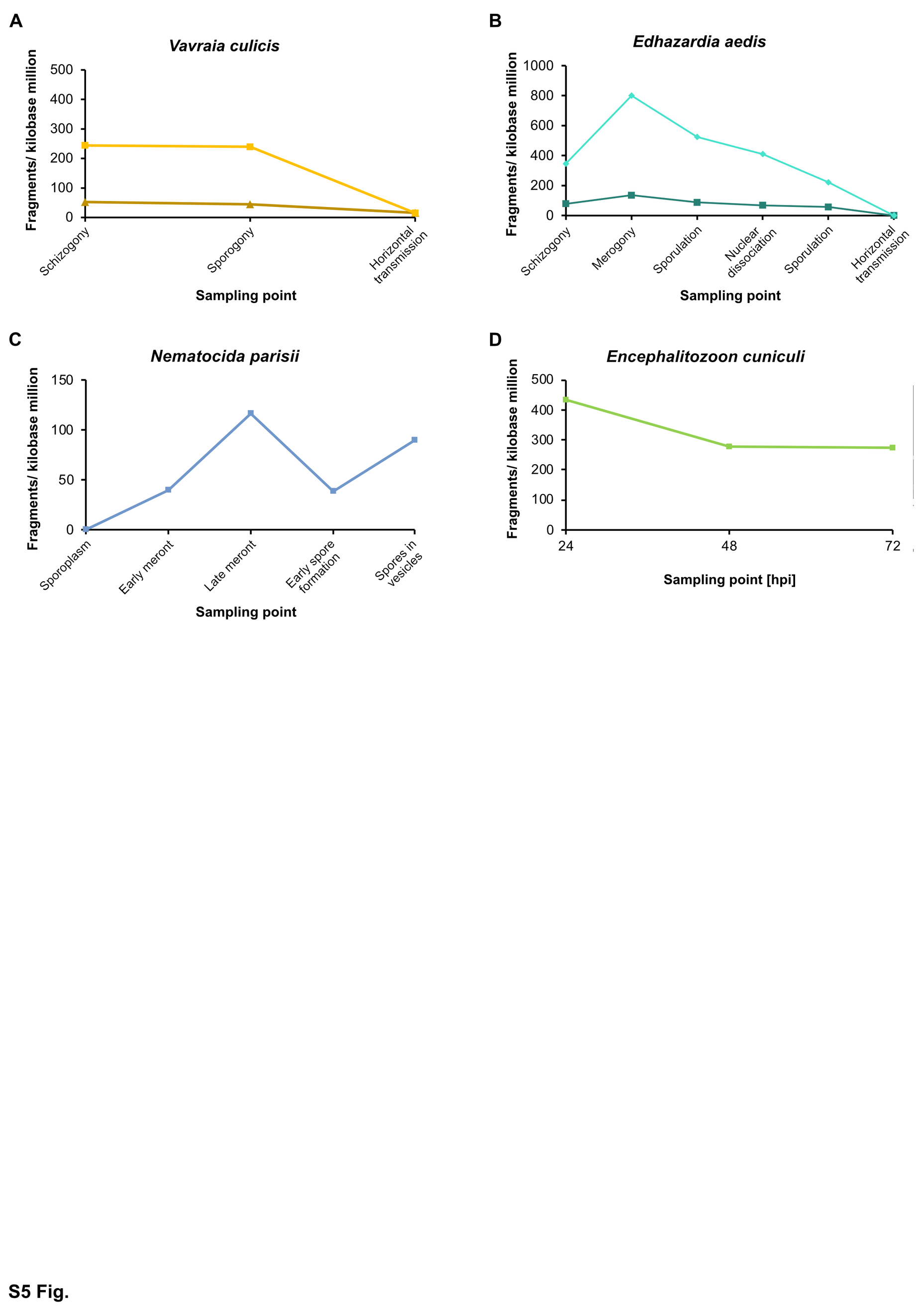

Supplement: S6 Fig — A,B) Gene expression pattern of mscS2 in two microsporidians, Vavraia culicis (A, VCUG_00285) with a simple, and Edhazardia aedis (A, EDEG_01910) with a complex life cycle [69]. The life-cycle stages corresponding to the sampling points are indicated. Each developmental stage was sampled in duplicates [69] and is shown in shades of yellow (A) and cyan (B). C) Transcript levels of Nematocida parisii mscS2 (NEPG_00116) over the course of a full infection cycle: 1) 8 hours post infection (hpi) sporoplasm stage, 2) 16 hpi early meront stage, 3) 30 hpi late meront stage, 4) 40 hpi onset of spore formation and 5) 64 hpi spores within membrane-bound vesicles. Samples from 8, 16, and 30 hpi are reported to be dominated by proliferating meronts, and later samples at 40 and 64 hpi harbor a mixture of meront, sporont and mature spore stages. Developmental stages were assessed by differential interference contrast microscopy and fluorescence in situ hybridization [1]. D) Transcriptional profile of mscS2 in Encephalitozoon cuniculi (Ecu09_0470) after 24, 48, and 72 hpi. Transcriptomic data indicates that at 24 hpi, proliferation rates are throttled due to the downregulation of housekeeping genes. Further, at 48 hpi, meronts start producing spore-related genes, but spore formation is not expected until after 72 hpi [70]. (TIF) [file pone.0301951.s006.tif]

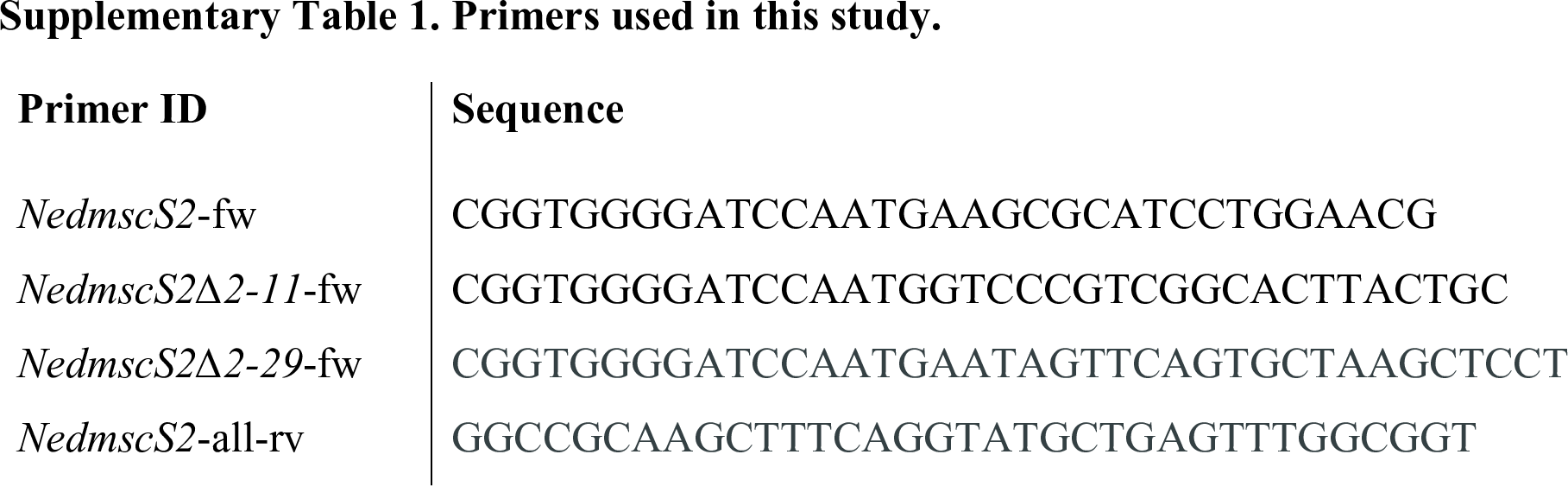

Supplement: S1 Table — (TIF) [file pone.0301951.s007.tif]

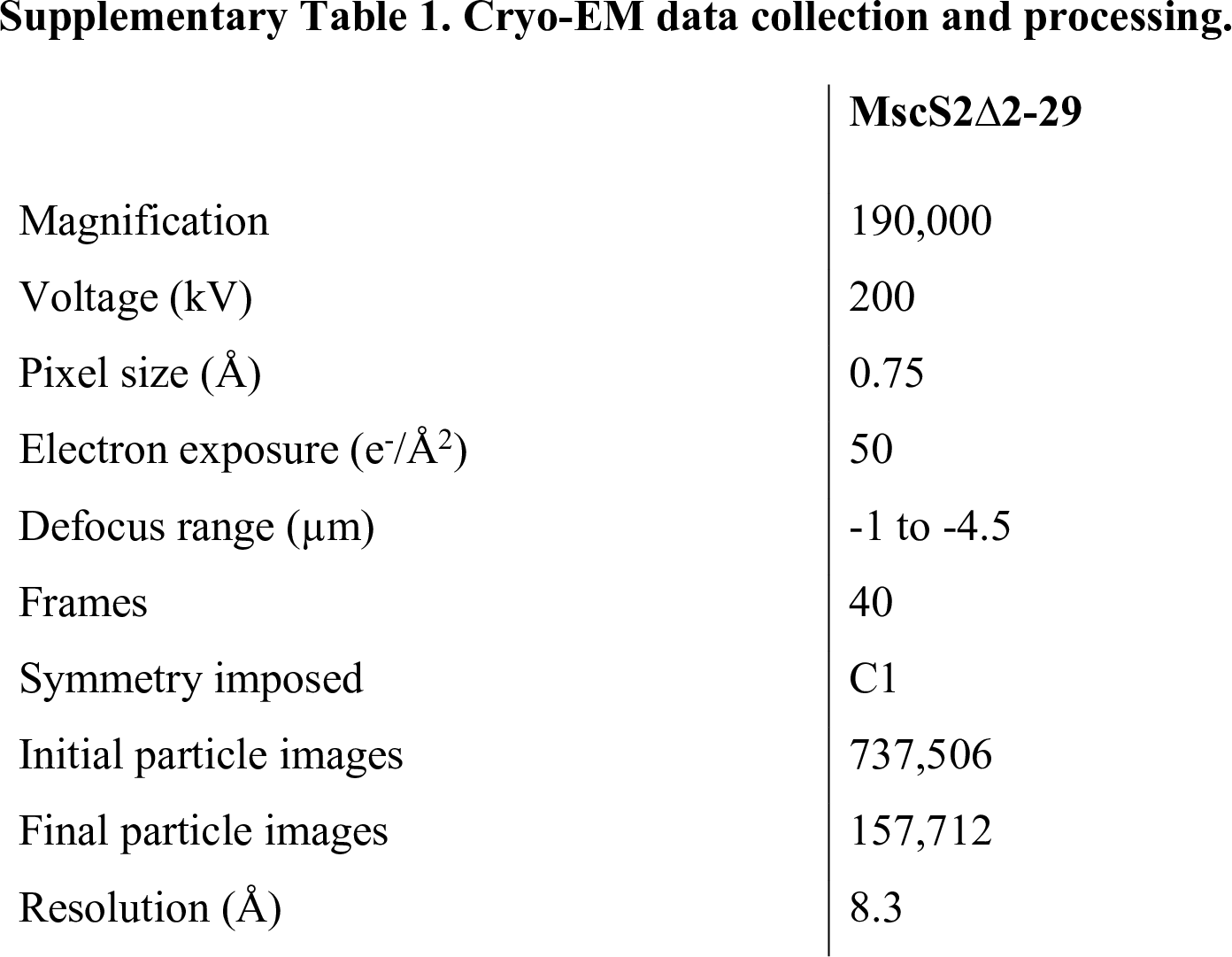

Supplement: S2 Table — (TIF) [file pone.0301951.s008.tif]
