# Supplementary material for: Nematocida displodere mechanosensitive ion channel of small conductance 2 assembles into a unique 6-channel super-structure in vitro
Source: PLoS One. 2024 Jul 22;19(7):e0301951. doi: 10.1371/journal.pone.0301951 (PMC11262690; doi:10.1371/journal.pone.0301951)
Supplement: Attachment — Submitted filename: Rebuttal.docx [file pone.0301951.s009.docx]

**Rebuttal**

We thank the two reviewers for reading our manuscript and providing constructive feedback. Especially reviewer one has given us valuable input that allowed us to improve the clarity of the manuscript. Below, we have addressed the comments point by point, provided our responses in blue font, and all changes in the main text and supplementary information are highlighted in cyan. We believe the manuscript has benefited from the suggested changes and hope that with these modifications, it can be accepted for publication in PLOS One.

**Reviewer #1:**

In this manuscript, Berg et al. study mechanosensitive ion channel of small conductance 2 (MscS2) from the microsporidium Nematocida displodere. Sequence analysis and AlphaFold modeling suggest that MscS2 lost most of the cytoplasmic domain of the archetypal MscS channel, retaining only the beta-domain, as well as most of the transmembrane domain, retaining possibly only a TM3-like helix, which forms the ion-conducting channel in MscS. After construct optimization, the authors purify an N-terminally truncated MscS2 from Nematocida displodere. Cryo-EM analysis yielded a density map at 8.3 Å resolution and showed that six MscS2 channels assemble into a superstructure resembling a six-way cross-joint. The authors interpret density for one of these channels using their AlphaFold model and conclude that MscS2 forms the expected heptamer. The authors further draw conclusions from the comparison of their 8.3 Å map with the structure of MscS, concluding that MscS2 is compacted compared to MscS. However, the authors recognize that - if MscS2 would indeed function as a mechanosensitive channel - the tension-sensing mechanism would have to be very different from that of MscS.

1. The study is technically done okay. However, the image processing that was performed is very basic and no advanced techniques, such as symmetry expansion*,* 3D variance analysis, masking of just one channel or substructure etc., were applied in order to obtain a higher-resolution map of the channel. I would expect more effort in image processing to be expended before publishing this rather low-resolution structure.

We appreciate that reviewer #1 recognized the technically satisfactory execution. Most of the suggestions related to data processing were already tried in an attempt to improve the resolution of the oligomeric assembly. While we omitted these trials in the first version of the manuscript, the revised version now includes a new Supplementary Figure S2, and a revised data processing section in the Materials and Methods outlining the challenges we faced.

However, we revisited the data processing again. This now included improved template picking, consecutive 2D classification and more thoroughly sorting out sub-optimal classes. This improved the resulting initial 3D models. We then performed 3D consensus refinement without symmetry and masked the 2 channels that looked best, which improved the volume. Now more features of the tertiary channel structure became visible, clearly showing that MscS2 is indeed a homo-heptamer.

We have updated the manuscript accordingly on lines 210-226 and lines 355-364. We also updated Supplementary Figure 1, added Supplementary Figure 2, and updated Figures 4 and 5.

2. The paper could also be presented much better. The introduction describes microsporidia in great detail, but this information is only needed for one supplementary figure (Fig. S5). On the other hand, the introduction lacks much of the known information on the structure and gating of MscS and, more importantly, does not discuss the many structural variations found in MscS-like channels. In fact, it is not at all clear whether MscS2 functions as a mechanosensitive channel, and it is structurally so different from MscS, especially the transmembrane domain, that it may be better to refer to MscS2 as an MscS-like channel rather than as an MscS channel. Basically, since this is a structural paper, the reader would be much better served if the introduction would provide more information on the structure of MscS and structural features of MscS

We agree with the reviewer that we should have included a better introduction to mechanosensitive channels in the manuscript. Additional information on the general structure and gating mechanisms of mechanosensitive channels, complemented with a new introductory figure (Figure 1) showing the open and closed state of *Escherichia coli* MscS, have now been added in lines 60-71.

Microsporidia still feature a major part of the introduction since the unusual compacted nature of the channel appears to be unique to microsporidia. However, we have now compacted the information.

Further, we agree with the reviewer that it is better to refer to MscS2 as a MscS-like channel and have modified the manuscript accordingly in lines 23, 27, 402-405, 414-419.

There are several points the authors have to address before this manuscript would be suitable for publication:

3. The authors conclude from their sequence and AlphaFold analyses that “with TMH3 and the conserved beta-domain, the microsporidian MscS2 maintains the essential domains of a functional mechanosensitive ion channel”. This conclusion is not justified. Most people consider helices TM1 and TM2 to be the tension-sensing module. Without these helices, it is not at all obvious that MscS2 could function as a mechanosensitive ion channel. To be able to state that MscS2 functions as a mechanosensitive (and not some other type of) ion channel, the authors would have to perform electrophysiology.

As stated previously, we agree with the reviewer that we cannot yet say whether MscS2 actually functions as a mechanosensitive ion channel or not. Since we are not in a position to perform any electrophysiological experiments, we have modified the text so that it is clear that the function of MscS2 remains to be defined. (See line changes in comment 2).

4. Figure 4 seems to be extremely misleading. It is very unusual to use such similar colors for the map and the model. It would be much more informative if the authors would use a high-contrast color for the model and a low-contrast color for the transparent surface.

We thank Reviewer #1 for pointing out the unfavorable color choice. We did not at all intend to be misleading, so we have now changed the colors of the model and adjusted the depth of focus for a clearer view (Figure 5A).

5. The authors also do not point out that the cryo-EM density (as an aside cryo-EM does not provide electron density - that would be X-ray diffraction) mostly just accounts for the cytoplasmic domain of MscS2 but not for any of the transmembrane helices.

We have now adjusted the text to make this clearer to the reader, in lines 381-383.

6. Another complication the authors do not address is that TM3a in MscS, despite its name, is actually not a transmembrane helix. It is in fact outside of the membrane, and it is in fact the ends of helices TM1 and TM2 that incorporate the channel into the membrane (the authors could indicate the membrane-spanning region for MscS and they will immediately notice the problem). Thus, the EcMscS 92-182 model shown in Fig. 4B would actually not have a transmembrane domain (if TM3a would be the same in MscS2 as in EcMscS). It seems very difficult to imagine that TM3a, which only forms the ion-conduction pathway (outside the membrane) in EcMscS, would serve the same purpose in MscS2 as well as have to incorporate into the membrane and also have sense membrane tension.

We agree with the reviewer that this could indeed be better described. In the updated manuscript, we added a new introductory figure (Figure 1) indicating the membrane, which is now also updated in the new Figure 5B. Further, we have modified the text accordingly in lines 397-405 and 414-416.

7. The authors discuss that MscS2 is greatly compacted compared to archetypal MscS and misses the tension-sensing helices, and so they conclude that MscS2 may sense tension differently from MscS2. However, given the substantially different transmembrane domain and since there are no functional data, it is not that far-fetched to assume that the function of MscS2 may actually have evolved away from being a mechanosensitive channel and may in fact NOT be a mechanosensitive channel at all. At this point, the function of MscS2 is not defined.

Reviewer #1 raises a valuable point. We address this in comment 2 above.

8. The mscS2 gene is predominantly upregulated during proliferative stages of microsporidia and is only moderately expressed during stages that require morphological changes (Fig S5). This seems to go against the idea that MscS2 functions mainly as a mechanosensitive channel regulating homeostasis, as the expected expression pattern would then be more like the opposite.

We thank Reviewer #1 for pointing this out and have removed the assumption that MscS2 acts as a homeostasis regulator in lines 435-436.

Minor points:

9. In the Abstract and Introduction, the authors write that microsporidia have at least 5 different mscS copies. However, the words “copies” and “different” are mutually exclusive because copies are, by definition, identical.

We thank Reviewer #1 for spotting this and have removed the specification in the abstract, as it is not relevant at this point. Regarding the introduction, the sentence was rephrased in line 77.

10. Figure 2B: It should be 28/56 “nM” not 28/56 “nm”.

We thank the reviewer for pointing this out and have corrected the units in Figure 2B.

11. Line 314: “MscS particles from E. coli visualized via TEM (56)”. The title of reference 56 is “Structural mechanism for gating of a eukaryotic mechanosensitive channel of small conductance”. E. coli is not a eukaryote, so this reference makes no sense.

We apologize for the incorrect reference here. We have now corrected the reference to *Zhang, Y., Daday, C., Gu, RX. et al. Visualization of the mechanosensitive ion channel MscS under membrane tension. Nature 590, 509–514 (2021).* [*https://doi.org/10.1038/s41586-021-03196-w*](https://doi.org/10.1038/s41586-021-03196-w)

and

*Catalano C, Ben-Hail D, Qiu W, Blount P, Des Georges A, Guo Y. Cryo-EM Structure of Mechanosensitive Channel YnaI Using SMA2000: Challenges and Opportunities. Membranes. 2021 Oct 31;11(11):849*

in line 337.

**Reviewer #2:**

Berg at al. describe an unusual oligomeric structure of a small variant of a mechanosensitive channel of small conductance. This channel lacks key elements of the prototypic bacterial MscS, such as a complete transmembrane sensor paddle and an enclosed cytosolic vestibule. The authors have used electron cryo microscopy for structure determination at intermediate resolution (sub-nanometer). The structure shows that 6 heptameric channels assembled into a cube-like super structure. This finding is unexpected and curious but probably without biological relevance. The authors relate the super structure to the C-terminal truncation of the rudimentary sensor paddle, which they introduced to improve expression and purification.

The manuscript is well written. It combines solid biochemical studies for establishing the oligomerization state, with electron microscopy for structure determination. The findings are complemented by sequence analysis for establishing the relation to other mechanosensitive channels.
To my knowledge, the authors present the 1st structure of a MscS-like channel with a reduced vestibule with an accessible pore. However, the modest resolution of the map is a missed opportunity for gaining more in depth understanding how ion selectivity is achieved in such a channel.

We thank Reviewer #2 for the positive feedback on the quality of the text, the biochemical analyses to assess the oligomeric state, and the bioinformatic analyses. We would have loved to provide a better-resolved structure, which we attempted to obtain early on in the project using different data processing methods, which we omitted for clarity reasons in the first version of the manuscript. Following both reviewer’s comments, we have now added a description of what we have done to improve the resolution in a new Supplementary Figure 2 and describe them in more detail in an updated materials and methods section. See also our response to comment 1 of the first reviewer.

In conclusion, we believe that the heptamers assemble in a heterogeneous manner, burying their hydrophobic N-termini in the middle of the assembly. The individual channels tilt slightly (no perfect symmetry – in C1 refinements, some channels are more tilted than others) and might be able to rotate along the central pore axis. The apparent heterogeneity did not allow for obtaining a high resolution.
